# Fenofibrate alleviates insulin resistance by reducing tissue inflammation in obese ovariectomized mice

**DOI:** 10.1038/s41387-023-00249-z

**Published:** 2023-11-07

**Authors:** Jungu Lee, Suyeon Jeon, Mijeong Lee, Michung Yoon

**Affiliations:** Department of Biological Sciences, Mokwon University, Daejeon, 35349 Korea

**Keywords:** Pre-diabetes, Obesity

## Abstract

**Background:**

Fenofibrate is a hypolipidemic peroxisome proliferator-activated receptor α (PPARα) agonist used clinically to reduce hypercholesterolemia and hypertriglyceridemia.

**Objective:**

We investigated the effects of fenofibrate on insulin resistance and tissue inflammation in a high-fat diet (HFD)-fed ovariectomized (OVX) C57BL/6J mice, a mouse model of obese postmenopausal women.

**Methods:**

Female OVX mice were randomly divided into 3 groups and received a low-fat diet, an HFD, or an HFD supplemented with 0.05% (w/w) fenofibrate for 9 weeks. Parameters of insulin resistance and tissue inflammation were measured using blood analysis, histological analysis, immunohistochemistry, and quantitative real-time polymerase chain reaction.

**Results:**

When fenofibrate was administered to HFD-fed OVX mice for 9 weeks, we observed reductions in body weight gain, adipose tissue mass, and the size of visceral adipocytes without the change of food intake. Fenofibrate improved mild hyperglycemia, severe hyperinsulinemia, and glucose tolerance in these mice. It also reduced pancreatic islet size and insulin-positive β-cell area to levels similar to those in OVX mice fed a low-fat diet. Concomitantly, administration of fenofibrate not only suppressed pancreatic lipid accumulation but also decreased CD68-positive macrophages in both the pancreas and visceral adipose tissue. Treatment with fenofibrate reduced tumor necrosis factor α (TNFα) mRNA levels in adipose tissue and lowered serum TNFα levels.

**Conclusion:**

These results suggest that fenofibrate treatment attenuates insulin resistance in part by reducing tissue inflammation and TNFα expression in HFD-fed OVX mice.

## Introduction

Fenofibrate has been used to reduce lipids in patients with hypercholesterolemia and hypertriglyceridemia. The effects of fenofibrate are mediated by nuclear peroxisome proliferator-activated receptor α (PPARα), which regulates the expression of genes critical for lipid and lipoprotein metabolism [1–3]. Our previous study showed that fenofibrate inhibits visceral obesity and adipocyte hypertrophy by stimulating fatty acid oxidation-related genes in obese mice [4–6]. Because visceral obesity and adipocyte hypertrophy are associated with insulin resistance [6–8], fenofibrate could potentially be effective in controlling insulin resistance.

Obesity is characterized by increases in both adipocyte size and the level of inflammation in adipose tissue. Markers of adipose tissue inflammation, such as increased infiltration of macrophages and higher expression of proinflammatory genes, are observed in the white adipose tissue of obese animals [9, 10]. Adipose tissue inflammation impairs insulin signaling and promotes the development of insulin resistance [11–13]. Higher numbers of macrophages infiltrate visceral adipose tissue than subcutaneous adipose tissue [14], suggesting that visceral obesity is closely associated with insulin resistance.

Chronic inflammation in adipose and liver is well known to be closely related to obesity. Recently, pancreatic islet inflammation has been also observed during the process of obesity. Islet-associated immune cells are increased in obese, diabetic animals and humans, where CD68-positive macrophages were the predominant immune cells [15–18]. For example, islets from high-fat diet (HFD)-fed C57BL/6J elevated the production and secretion of inflammatory factors [15] and islet macrophages were correlated with hyperglycemia, glucose intolerance, and homeostasis model assessment insulin resistance (HOMA-IR) [19]. Thus, pancreatic islet inflammation may negatively affect glucose metabolism.

Postmenopausal women are easily exposed to weight gain and metabolic diseases such as hypertension, hyperlipidemia, diabetes, and insulin resistance [20, 21]. Similar to postmenopausal women, ovariectomized (OVX) mice also have high rates of obesity and impaired glucose tolerance [22]. Obese OVX mice are animal models used to study obesity and its metabolic diseases in postmenopausal women. Accordingly, we hypothesized that fenofibrate modulates obesity-related insulin resistance by decreasing tissue inflammation in HFD-fed obese OVX mice.

The aims of this study were to determine whether fenofibrate inhibits visceral obesity, hyperglycemia, and insulin resistance in HFD-fed obese OVX mice and to investigate the mechanism of action of fenofibrate. Here, we show that fenofibrate normalizes the elevated level of circulating glucose and attenuates insulin resistance by reducing inflammation in adipose tissue and pancreas of HFD-fed OVX mice.

## Methods

### Animal treatments

Female wild-type C57BL/6J mice (*n* = 5/group) were purchased from Central Lab Animal (Seoul, Korea). After 8-week-old mice were OVX, animals were randomly divided into three groups and received a low-fat diet (LFD, 13 kcal% fat, Research Diets, New Brunswick, NJ, USA), an HFD (45 kcal% fat, Research Diets), or an HFD supplemented with 0.05% (w/w) fenofibrate (HFD-FF) for 9 weeks. The body weight of each mouse was measured three times a week by a researcher blinded to each experimental group. On the last day of the study, 8-h-fasted mice were killed by cervical dislocation. All mouse studies were approved by the Institutional Animal Care and Use Committees of Mokwon University (permit number: NVRQS AEC-18), and performed according to the ARRIVE guidelines.

### Blood analysis

Serum levels of alanine aminotransferase (ALT), aspartate aminotransferase (AST), and triglycerides were quantified using a blood chemical analyzer (Cobas 8000, c502, Roche, Grenzach-Wyhlen, Germany). Serum levels of free fatty acids were analyzed using SICDIA NEFAZYME (Shinyang, Seoul, Korea). Blood glucose levels were determined using the Accu-Chek Performa System (Roche, Basel, Switzerland). Serum insulin and TNFα levels were measured using a Rat/Mouse Insulin ELISA Kit (EZRMI-13K, Millipore, Burlington, MA, USA) and a Mouse TNFα Quantikine HS ELISA Kit (MHSTA50, McKinley Pl NE, MN, USA), respectively. Oral glucose tolerance tests were performed with 2 g/kg body weight. Quantitative insulin sensitivity check index (QUICKI) values were calculated as follows: 1/(log (fasting insulin μU/mL) + log (fasting glucose mg/dL)). HOMA-IR was calculated via an online Oxford HOMA calculator (available at: www.dtu.ox.ac.uk) using the formula: (fasting insulin μU/mL × fasting glucose mg/dL)/405.

### Histological analysis

Pancreata and adipose tissues were fixed in 10% formalin for 1 day and embedded in a paraffin block. Pancreas and parametrial adipose tissue sections (5 μm) were cut and stained with hematoxylin-eosin (HE). Stained sections were analyzed under an Olympus BH2-RFCA fluorescence microscope (Olympus, Tokyo, Japan) and estimated with an image analysis system (ImageJ software, http://imagej.nih.gow/ij/).

### Immunohistochemistry

Pancreata and adipose tissues were fixed in 10% formalin and embedded in a paraffin block. After epitope retrieval, pancreas sections (3-μm thick) were stained with anti-insulin (1:1400 dilution; I2018; Sigma-Aldrich, St Louis, MO, USA) or anti-CD68 (1:200 dilution; ab955; Abcam, Cambridge, UK) primary antibodies and with an anti-mouse IgG biotinylated secondary antibody (Vector Laboratories, Burlingame, CA, USA). Parametrial adipose tissues (3 μm thick) were stained with an anti-CD68 antibody and were counterstained with Mayer’s hematoxylin. Immunostained sections were evaluated using ImageJ software.

### Quantitative real-time polymerase chain reaction (PCR)

Total RNA was extracted from visceral adipose tissues using Trizol reagent (Invitrogen, Carlsbad, CA, USA). cDNA was prepared from 2 μg total RNA using the TOPscript^TM^ DryMIX reverse transcription kit (Enzynomics, Seoul, Korea). Real-time PCR was performed using iQ SYBR Green Supermix (Bio-Rad, Hercules, CA, USA) and a Excycler^TM^ 96 Real-Time Quantitative Thermal Block machine (Bioneer, Daejeon, Korea). The primer sequences were as follows: tumor necrosis factor α (TNFα) (forward 5’-GCTGAGCTCAAACCCTGGTA-3’, reverse 5’-CTGAGTTGGTCCCCCTTCTC-3’) and β-actin (forward 5’-TGGAATCCTGTGGATCCAT-3’, reverse 5’TGGTACCACCAGACAGCACTG-3’). PCR was performed using the following conditions: 1 cycle of 95 °C for 5 min, followed by 40 cycles of 95 °C for 10 s and 55 °C for 20 s. TNFα mRNA levels were normalized using β-actin.

### Statistical analysis

The researchers were blinded to the group assignment during the experiment and/or when evaluating the results. The sample size of each experiment was determined based on previous studies using mouse models of visceral obesity and insulin resistance. The exact sample size (*n*) for each experimental group is shown in the figure legend. The animals were randomly assigned to the experimental group and processed. Values were expressed as mean ± standard deviation (SD). Statistical analysis was performed using analysis of variance followed by Turkey’s post-hoc tests. Statistical significance was defined as *p* < 0.05.

## Results

### Fenofibrate regulates visceral obesity and adipocyte hypertrophy in obese OVX mice

**Fig. 1:**
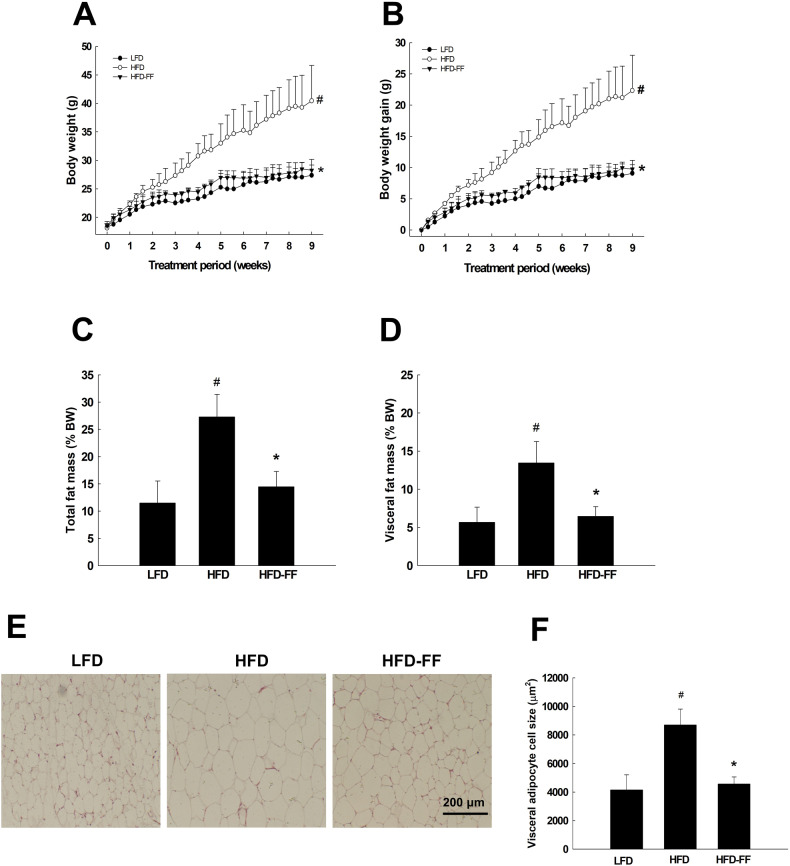
Body weight, adipose tissue mass, and visceral adipocyte size. Ovariectomized mice (*n* = 5/group) were fed an LFD, an HFD, or an HFD-FF for 9 weeks. **A** Body weights and **B** body weight gains at the end of the treatment period are significantly different between the HFD group and the LFD or HFD-FF (*p* < 0.05) groups. **C** Total and **D** visceral adipose tissues (% body weight). **E** Hematoxylin & eosin-stained sections of visceral adipose tissue (original magnification x100). **F** Visceral adipocyte size. All values are expressed as the mean ± SD. ^#^*p* < 0.05 compared with LFD. *p < 0.05 compared with HFD.

Average body weight and body weight gain were measured in OVX mice maintained for 9 weeks on HFD supplemented with fenofibrate (HFD-FF mice) or HFD without fenofibrate (HFD mice). The results revealed a 30% reduction in body weight and a 56% reduction in body weight gain for HFD-FF mice than for HFD mice (*p* < 0.05; Fig. 1A, B). Treatment with fenofibrate also significantly decreased total and visceral adipose tissue weights by 64% and 67%, respectively, in HFD-FF mice compared with HFD mice (Fig. 1C, D). Histological analysis of adipose tissue showed that fenofibrate treatment decreased the sizes of visceral adipocytes, such that the mean size in HFD-FF mice was 46% smaller than the mean size in HFD mice (Fig. 1E, F). However, there was no significant difference in food intake between the HFD and HFD-FF mice (data not shown).

### Fenofibrate lowers circulating glucose levels by alleviating insulin resistance in obese OVX mice

**Fig. 2:**
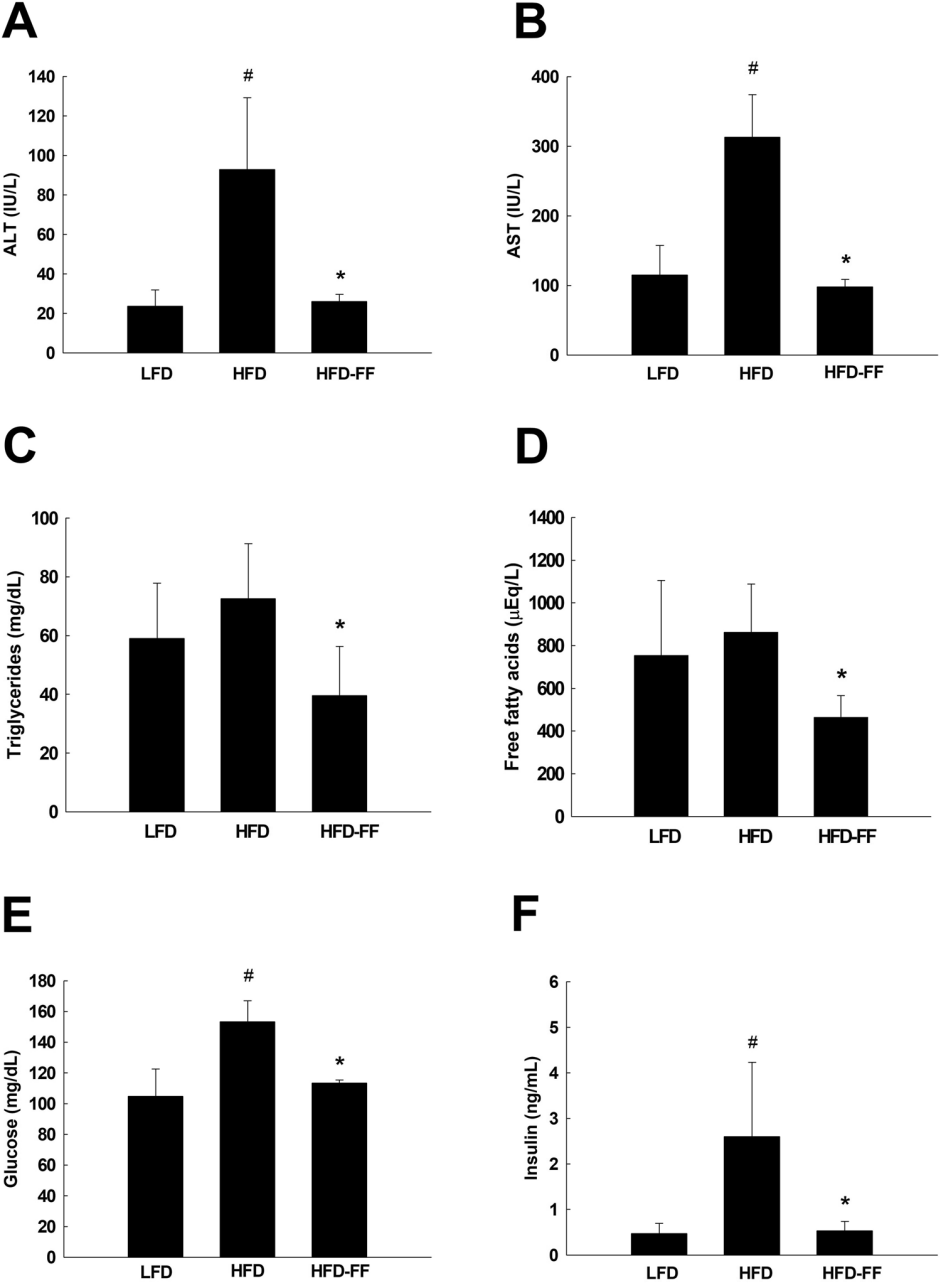
Circulating levels of ALT, AST, lipids, glucose, and insulin. Ovariectomized mice (*n* = 5/group) were fed an LFD, an HFD, or an HFD-FF for 9 weeks. Serum levels of **A** ALT, **B** AST, **C** triglycerides, and **D** free fatty acids. **E** Fasting blood glucose and **F** serum insulin levels. All values are expressed as the mean ± SD. ^#^*p* < 0.05 compared with LFD. *p < 0.05 compared with HFD.

**Fig. 3:**
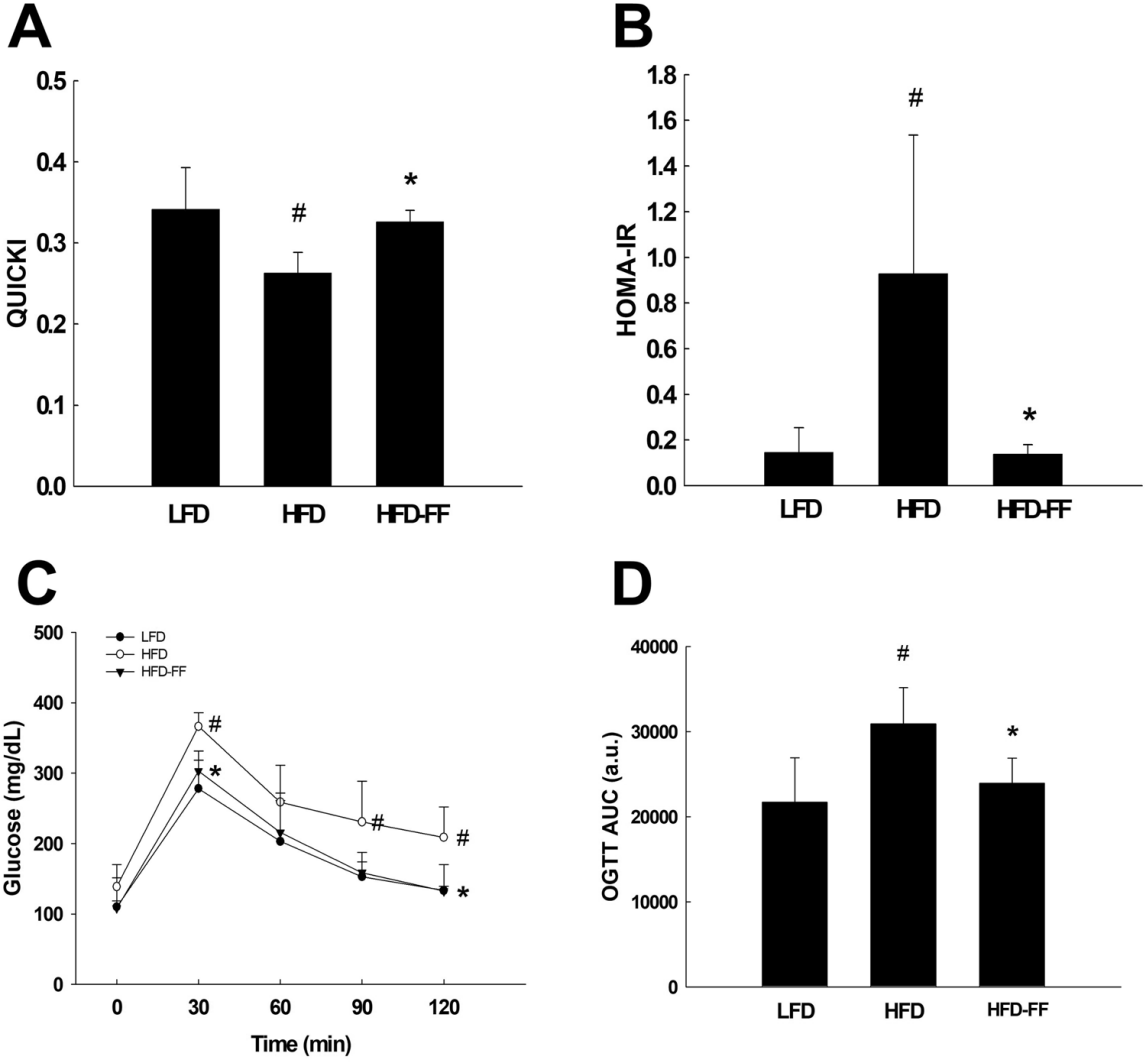
QUICKI, HOMA-IR, and OGTT. Ovariectomized mice (*n* = 5/group) were fed an LFD, an HFD, or an HFD-FF for 9 weeks. **A** QUICKI and **B** HOMA-IR. **C** OGTT and **D** OGTT area under the curve (AUC). All values are expressed as the mean ± SD. ^#^*p* < 0.05 compared with LFD. *p < 0.05 compared with HFD.

HFD-FF mice demonstrated significantly lower levels of serum ALT, AST, triglycerides, and free fatty acids than untreated HFD mice (Fig. 2A–D). Relative to LFD mice, fasting blood glucose and serum insulin levels were 47% and 453% higher, respectively, in HFD mice, showing that HFD induced mild hyperglycemia and severe hyperinsulinemia in OVX mice. By contrast, fasting blood glucose and serum insulin levels were 26% and 80% lower, respectively, in HFD-FF mice than in HFD mice (Fig. 2E, F). Insulin sensitivity was higher in HFD-FF mice than in HFD mice when measured with the accurate QUICKI index (Fig. 3A). Quantification using the HOMA-IR score suggested that insulin resistance was lower in HFD-FF mice than in HFD mice (Fig. 3B). Similarly, an oral glucose tolerance test (OGTT) performed 30 or 120 min after glucose administration revealed lower blood glucose levels in HFD-FF mice than in HFD mice (Fig. 3C, D).

### Fenofibrate normalizes islet β-cell mass in obese OVX mice

**Fig. 4:**
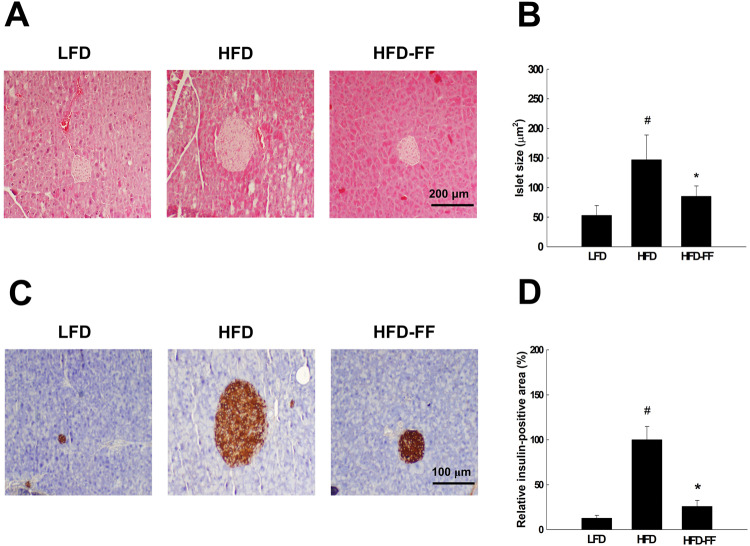
Pancreatic islet morphology and β-cell mass. Ovariectomized mice (*n* = 5/group) were fed an LFD, an HFD, or an HFD-FF for 9 weeks. **A** Hematoxylin & eosin-stained pancreas section (original magnification x100). **B** Mean size of islets (μm^2^). **C** Pancreas sections stained with an anti-insulin antibody (original magnification x200). **D** Relative insulin-positive area. All values are expressed as the mean ± SD. ^#^*p* < 0.05 compared with LFD. *p < 0.05 compared with HFD.

Pancreatic islets were 177% larger in HFD mice than in LFD-fed OVX mice (LFD mice; Fig. 4A, B). However, pancreatic islets were 42% smaller in HFD-FF mice than in HFD mice. In addition, insulin-positive β-cell area and islet β-cell mass were 691% higher in HFD mice than in LFD mice (Fig. 4C, D), whereas the β-cell areas in HFD-FF mice were similar to those in LFD mice and reduced by 74%. Overall, fenofibrate reduced pancreatic islet size and insulin-positive β-cell area to levels similar to those in LFD mice.

### Fenofibrate inhibits pancreatic steatosis and inflammation in obese OVX mice

**Fig. 5:**
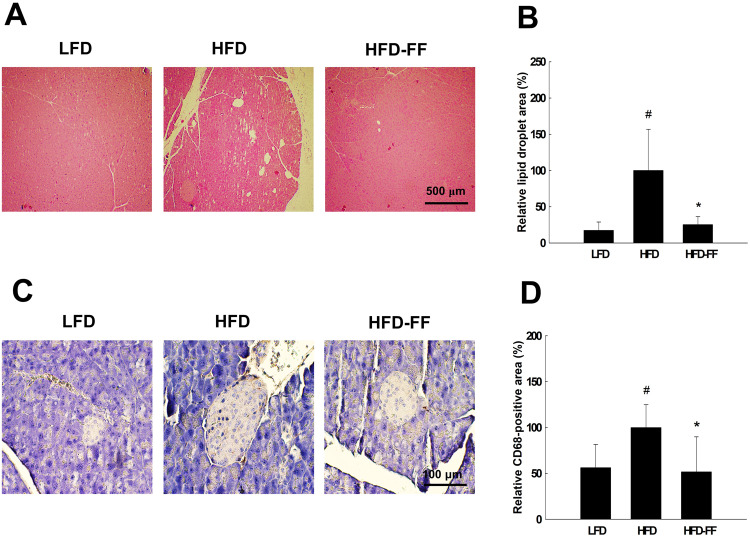
Pancreatic steatosis and inflammation. Ovariectomized mice (*n* = 5/group) were fed an LFD, an HFD, or an HFD-FF for 9 weeks. **A** Hematoxylin & eosin-stained pancreas section (original magnification x40). **B** Relative lipid droplet area. **C** Pancreas sections stained with an antibody against CD68 (original magnification x200). **D** Relative CD68-positive area. All values are expressed as the mean ± SD. ^#^*p* < 0.05 compared with LFD. *p < 0.05 compared with HFD.

We observed pancreatic steatosis in HFD mice, indicated by an increase in lipid droplets relative to LFD mice, but the treatment of HFD mice with fenofibrate reversed this effect and decreased lipid droplets (Fig. 5A, B). HFD mice had 77% higher levels of pancreatic inflammation than LFD mice, as revealed by an increase in the area of CD68-positive cells (Fig. 5C, D). Fenofibrate treatment of HFD mice also suppressed this effect, reducing the area of CD68-positive cells in the pancreas by 48%.

### Fenofibrate suppresses adipose tissue inflammation and serum TNFα levels in obese OVX mice

**Fig. 6:**
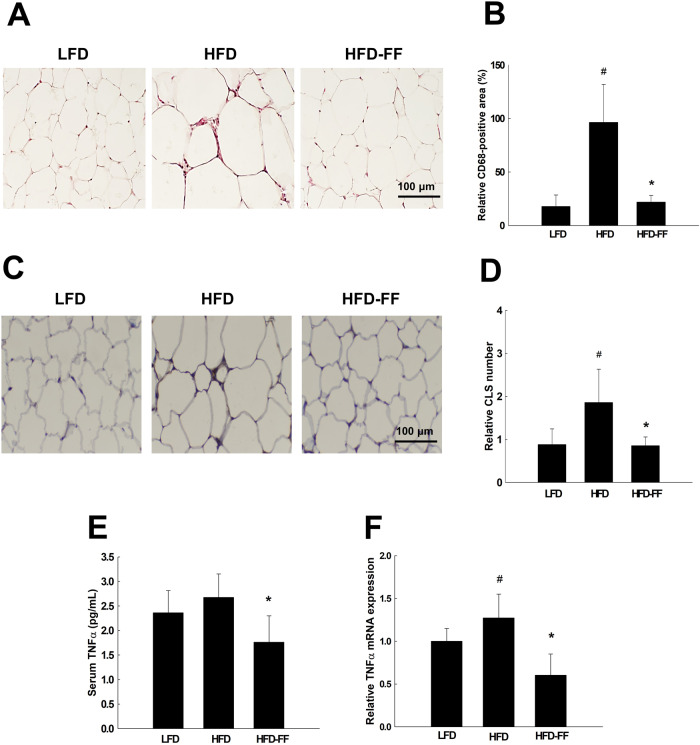
Visceral adipose tissue inflammation and serum levels and adipose tissue expression of TNFα. Ovariectomized mice (*n* = 5/group) were fed an LFD, an HFD, or an HFD-FF for 9 weeks. **A** Visceral adipose tissue section stained with an antibody against CD68 (original magnification x200). **B** Relative CD68-positive area. **C** Hematoxylin & eosin-stained sections of visceral adipose tissue showing CLS (original magnification x100). **D** Relative CLS number. **E** Serum levels and **F** adipose tissue expression of TNFα. All values are expressed as the mean ± SD. ^#^*p* < 0.05 compared with LFD. *p < 0.05 compared with HFD.

The abundances of CD68-positive macrophages and crown-like structures (CLSs) in visceral adipose tissue were 463% and 111% higher in HFD mice than in LFD mice, respectively (Fig. 6A–D). However, treatment of HFD mice with fenofibrate reduced the abundance of CD68-positive cells and CLSs by 78% and 54%, respectively, relative to untreated HFD mice. Fenofibrate treatment in HFD mice also reduced serum TNFα levels by 34% and decreased adipose tissue TNFα mRNA by 53% relative to untreated HFD mice (Fig. 6E, F).

## Discussion

Metabolic syndrome is a clinical phenotype characterized by visceral obesity, insulin resistance, dyslipidemia, and hypertension and has a higher prevalence in postmenopausal women than in premenopausal women [20, 21]. Our previous studies have demonstrated that fenofibrate regulates obesity and lipid metabolism in male mice, but not in female mice, indicating the sexual dimorphism of fenofibrate in obesity [4]. In female mice with healthy ovaries, estrogen appears to inhibit PPARα function by interfering with the DNA binding of PPARα [23]. Therefore, it is meaningful to expect that fenofibrate-induced weight gain and reduction in adipose tissue mass in male mice would be observed in OVX female mice.

HFD-fed C57BL/6 mice develop obesity and associated characteristics that resemble metabolic syndrome in humans [24]. In our study, after 9 weeks on HFD, OVX C57BL/6 J mice showed increases in body weight, body weight gain, and fat mass, demonstrating that HFD-fed OVX mice are suitable as a mouse model of obese postmenopausal women. However, fenofibrate treatment suppressed visceral obesity in HFD mice. Animals with HFD-induced obesity began to lose weight after 2 weeks of treatment with fenofibrate. The weights of total and visceral adipose tissue in HFD-FF mice also decreased significantly, approaching the levels in LFD mice. Consistent with the observed changes in visceral adipose tissue weight, the size of visceral adipocytes was 46% smaller on average in HFD-FF mice than in HFD mice, indicating that fenofibrate inhibits adipocyte hypertrophy, a notable feature of obesity. Furthermore, treatment with fenofibrate led to more prominent reductions in obesity-related parameters in HFD-fed OVX mice than in HFD-fed male mice, whereas these reductions were not observed in female HFD-fed mice with functioning ovaries [4, 25]. Fenofibrate has been shown to modulate mouse obesity by enhancing hepatic fatty acid oxidation and reducing circulating triglyceride levels, which are responsible for adipocyte hypertrophy [4–6]. Therefore, fenofibrate-activated PPARα is suggested to be involved in the regulation of obesity-induced insulin resistance.

In addition to adipocyte hypertrophy, another feature of obesity is increased adipose tissue inflammation, characterized by increased macrophage infiltration into adipose tissue and increased expression of proinflammatory genes [26–28]. Macrophages infiltrate adipose tissue at higher numbers in obese animals and humans than in non-obese animals and humans [9, 10, 29]. Macrophages can represent up to 40% of all cells in adipose tissue in obese animals [10, 30]. Macrophages surround hypertrophic adipocytes in the adipose tissue of obese mice and humans, resulting in the formation of CLSs [28]. We observed inflammation in the visceral adipose tissue of obese OVX mice, evidenced by increases in CD68-positive macrophages and CLS formation, and treatment with fenofibrate reduced both. Macrophages in visceral adipose tissue also secrete several proinflammatory factors such as TNFα, monocyte chemoattractant protein 1 (MCP-1), and interleukin (IL)-1β, which have been implicated in the development of insulin resistance [10, 28, 31]. Macrophages infiltrating adipose tissue in obese humans and animals produce large amounts of TNFα and MCP-1 [29, 32, 33]. In this study, HFD mice exhibited higher levels of adipose tissue TNFα mRNA and serum TNFα, both of which were reduced by treatment with fenofibrate. These results suggest that fenofibrate may mitigate insulin resistance by decreasing macrophage accumulation and TNFα expression in visceral adipose tissues of obese OVX mice. TNFα is an abundant proinflammatory cytokine found in the adipose tissue of obese, diabetic rodents [32]. In addition, neutralization of TNFα significantly increased insulin-stimulated peripheral uptake of glucose [34, 35], a result that demonstrates a strong association between higher TNFα levels and insulin resistance. Recently, it has been shown that aleglitazar, a PPARα/γ dual agonist, restored glucose uptake by decreasing TNFα-mediated inhibition of insulin-stimulated Akt (Ser473) phosphorylation and reducing TNFα–induced insulin receptor substrate 1 (Ser312) phosphorylation in insulin-resistant human adipocytes [36–38]. Thus, the PPARα activator fenofibrate is also likely to promote insulin signaling through inhibition of TNFα in visceral adipose tissue of HFD mice.

Visceral adipocyte hypertrophy and visceral adipose tissue inflammation are strong predictors of insulin resistance [9, 27, 39], which is consistent with the detection of adipocyte hypertrophy, adipose tissue inflammation, increased fasting glucose, and increased levels of insulin in HFD mice in the present study. In healthy, non-diabetic female C57BL/6 J mice, fasting blood glucose and plasma insulin levels are often measured at 80–100 mg/dL after fasting for 4–6 h and at 0.05–0.26 ng/mL after a 5 h fast, respectively, (http://www.jax.org/phenome), even though normal ranges of glucose and insulin levels in the blood vary depending on the length of the fast. We observed 26% (HFD vs. HFD-FF: 153 ± 13 vs. 113 ± 2 mg/dL) and 80% (HFD vs. HFD-FF: 2.6 ± 1.4 vs. 0.53 ± 0.2 ng/mL) reductions in circulating glucose and insulin levels after an 8-h fast, respectively, indicating hypoglycemic and hypoinsulinemic effects of fenofibrate in HFD mice. The level of circulating glucose also decreased during oral glucose tolerance testing of HFD-FF mice, demonstrating that fenofibrate alleviates impaired glucose tolerance in obese OVX mice. In addition, insulin sensitivity and insulin resistance were quantified using QUICKI and HOMA-IR, respectively, which are both based on fasting levels of circulating glucose and insulin [40, 41]. HFD mice had higher HOMA-IR and lower QUICKI values than LFD mice, and fenofibrate reversed the effects of HFD-feeding on these indices. Hence, fenofibrate may inhibit the development of obesity-induced hyperglycemia and insulin resistance in OVX mice.

Pancreatic β-cell proliferation leads to higher levels of insulin, preventing hyperglycemia associated with insulin resistance and maintaining normoglycemia through a process known as β-cell compensation [42, 43]. When β-cell compensation for insulin resistance is inadequate, impaired glucose tolerance may occur. Female HFD-fed mice show β-cell mass expansion and pancreatic islet hypertrophy [44]. In the present study, we observed that pancreatic β-cell mass increased and pancreatic islets exhibited hypertrophy in HFD mice. However, since fenofibrate treatment reduced islet size and decreased insulin-secreting β-cell areas in HFD mice to levels similar to those in LFD mice, normalization of β-cell mass by fenofibrate is likely to lower circulating insulin levels and suppress insulin resistance during obesity in OVX mice.

In the obese state, ectopic fat deposits are frequently observed in peripheral tissues, such as the liver, skeletal muscle, and pancreas, leading to hyperinsulinemia and insulin resistance [45–47]. The accumulation of pancreatic fat, known as fatty pancreas or pancreatic steatosis, is a phenomenon associated with the infiltration of adipocytes into the pancreas and increased intracellular lipid droplets [48, 49]. Although pancreatic lipids accumulated in HFD mice, fenofibrate treatment decreased obesity-induced deposition of pancreatic fat and inhibited pancreatic steatosis. Infiltration of adipocytes into the pancreas is associated with the infiltration of macrophages into or around pancreatic islets, as shown by an increased number of CD68-positive cells per islet [19]. The number of immune cells also increases in the pancreatic islets of obese mice, such as HFD-fed C57BL/6J and *db/db* mice [15]. Islets isolated from obese mice increased the production and release of inflammatory factors including IL-6, IL-8, IL-1β, and macrophage inflammatory protein 1α [15, 50]. Islet macrophages also contribute to islet β-cell proliferation and hyperplasia by producing these proinflammatory cytokines and platelet-derived growth factors during the process of obesity [17, 51, 52]. We observed that the number of pancreatic CD68-positive macrophages was higher in HFD mice than in LFD mice, whereas fenofibrate treatment inhibited the infiltration of macrophages in the pancreas. Because pancreatic steatosis and inflammation are correlated with HOMA-IR and glucose intolerance and appear to exacerbate hyperglycemia [18, 19, 53, 54], fenofibrate may alleviate glucose intolerance and insulin resistance by suppressing the accumulation of pancreatic fat and the infiltration of macrophages.

In conclusion, these results demonstrate that fenofibrate improves insulin resistance, in part by modulating inflammation in the adipose tissue and pancreas of obese OVX mice. Thus, fenofibrate may represent a potential treatment for obesity and insulin resistance in postmenopausal women.

## Data Availability

The data of this study are available from the corresponding author upon reasonable request.
